# Variation in growth rate, carbon assimilation, and photosynthetic efficiency in response to nitrogen source and concentration in phytoplankton isolated from upper San Francisco Bay

**DOI:** 10.1111/jpy.12535

**Published:** 2017-05-02

**Authors:** Gry Mine Berg, Sara Driscoll, Kendra Hayashi, Melissa Ross, Raphael Kudela

**Affiliations:** ^1^ Applied Marine Sciences 911 Center Street Santa Cruz California 95060 USA; ^2^ Ocean Sciences Department University of California 1156 High Street Santa Cruz California 95064 USA

**Keywords:** ammonium tolerance, carbon assimilation, chlorophytes, diatoms, growth rates, nitrogen source, PSII efficiency, upper San Francisco Bay

## Abstract

Six species of phytoplankton recently isolated from upper San Francisco Bay were tested for their sensitivity to growth inhibition by ammonium (NH_4_^+^), and for differences in growth rates according to inorganic nitrogen (N) growth source. The quantum yield of photosystem II (F_v_/F_m_) was a sensitive indicator of NH_4_^+^ toxicity, manifested by a suppression of F_v_/F_m_ in a dose‐dependent manner. Two chlorophytes were the least sensitive to NH_4_^+^ inhibition, at concentrations of >3,000 μmoles NH_4_^+^ · L^−1^, followed by two estuarine diatoms that were sensitive at concentrations >1,000 μmoles NH_4_^+^ · L^−1^, followed lastly by two freshwater diatoms that were sensitive at concentrations between 200 and 500 μmoles NH_4_^+^ · L^−1^. At non‐inhibiting concentrations of NH_4_^+^, the freshwater diatom species grew fastest, followed by the estuarine diatoms, while the chlorophytes grew slowest. Variations in growth rates with N source did not follow taxonomic divisions. Of the two chlorophytes, one grew significantly faster on nitrate (NO_3_^−^), whereas the other grew significantly faster on NH_4_^+^. All four diatoms tested grew faster on NH_4_^+^ compared with NO_3_^−^. We showed that in cases where growth rates were faster on NH_4_^+^ than they were on NO_3_^−^, the difference was not larger for chlorophytes compared with diatoms. This holds true for comparisons across a number of culture investigations suggesting that diatoms as a group will not be at a competitive disadvantage under natural conditions when NH_4_^+^ dominates the total N pool and they will also not have a growth advantage when NO_3_^−^ is dominant, as long as N concentrations are sufficient.

**Abbreviations**
*C*_0_: starting cell abundance
C: carbon
*C*: cell abundance
Chl *a*: chlorophyll *a*
DIC: dissolved inorganic carbon
EC_50_: 50% decrease in growth rate
F_0_: background Chl *a* fluorescence
F_m_: maximal Chl *a* fluorescence
F_v_/F_m_: quantum yield of photosystem II
F_v_: variable fluorescence
*k*: growth constant
LED: light‐emitting diode
N:P: nitrogen:phosphorus
NH_3_: ammonia
NH_4_^+^: ammonium
N: nitrogen
NO_3_^−^: nitrate
PAM: pulse‐amplitude‐modulated
PSII: photosystem II
*t*: time

Seasonally high NO_3_^−^ concentrations drive primary productivity and biomass accumulation in coastal and freshwater systems world‐wide (Sieracki et al. 1993, Malone et al. 1996, Collos et al. 1997, Berg et al. 2001, Kristiansen et al. 2001). However, in some coastal systems subjected to concentrated inputs of wastewater effluent, NH_4_^+^ has become an equally important, and at times even a dominant, N source. For example, NH_4_^+^ concentrations have increased dramatically in Lake Taihu, China (Chen et al. 2003), Deep Bay and Victoria Harbor, Hong Kong (Xu et al. 2010, 2011), and Colne Estuary, UK (Underwood and Provot 2000), to mention a few. Recent reports of seasonal succession of phytoplankton with changes in the dominant N source have questioned whether diatoms may be more competitive vis‐a‐vis other members of the phytoplankton community at times when NO_3_^−^ dominates the total N pool compared to when NH_4_^+^ does (Berg et al. 2003, Heil et al. 2007). As a result, it has been predicted that in systems with increased inputs of wastewater effluent, phytoplankton community composition may become skewed away from diatoms (Glibert et al. 2011). Confounding investigations into the effect of changes in N sources on phytoplankton succession is that total N concentrations typically change concomitantly, making it difficult to separate the effect of changes in N species from change in total N concentration (Berg et al. 2003, Flynn 2010, Davidson et al. 2012).

Suisun Bay, situated in the northern region of San Francisco Bay, California (Fig. S1 in the Supporting Information), receives elevated inputs of nutrients from the Sacramento River and is dominated by diatoms, making it an ideal system to investigate the nitrogenous nutrition of diatoms. While diatoms comprise the principal fraction of the phytoplankton community in Suisun Bay, their biomass has decreased over the last two decades (Alpine and Cloern 1992, Lehman 1996, 2000, Jassby 2008). Among the many hypotheses advanced to explain the decline in phytoplankton standing stocks is a change in the dominance of N species from NO_3_^−^ to NH_4_^+^. It has been hypothesized that NH_4_^+^ inhibits diatom growth and spring bloom formation at concentrations of 4 μmol · L^−1^ or greater (Dugdale et al. 2007). In contrast, chlorophytes and flagellates are hypothesized not be sensitive to NH_4_^+^ at the same low concentrations and therefore will not experience the same levels of growth inhibition (Glibert et al. 2011).

To test the hypothesis that diatoms have a low tolerance for NH_4_^+^, and grow faster when using NO_3_^−^ compared with NH_4_^+^ as a source of N for growth, we isolated a number of diatom and non‐diatom taxa directly from Suisun Bay and the Sacramento River into pure culture. This avoided several of the confounding factors with field investigations, including using a mixed plankton community as well as the difficulty of separating the effect of a change in the type of N (NO_3_^−^ vs. NH_4_^+^), from a change in the absolute N concentration. It also provided standardization for all other factors, including light, temperature, and base media composition. In addition, using freshly isolated strains rather than strains from culture collections avoided issues related to genetic adaptations from growing at unnaturally high N concentrations for many decades (e.g., Lakeman et al. 2009), and issues with extrapolation of results using strains isolated from other geographic regions to our particular locale.

The specific questions we asked were: (i) Do diatoms grow faster when using NO_3_^−^ compared with using NH_4_^+^ as the sole source of N? (ii) Do non‐diatoms grow faster when using NH_4_^+^ compared with using NO_3_^−^ as the sole source of N? (iii) Are lower growth rates on NH_4_^+^ the result of NH_4_^+^ inhibition or toxicity? (iv) If so, what are the levels of NH_4_^+^ that will result in a 50% decrease in phytoplankton growth rate (i.e., EC_50_)? The EC_50_ is commonly used in ecotoxicological studies as the benchmark of growth inhibition, and has also been applied with respect to inhibition of phytoplankton growth by NH_4_^+^ (Collos and Harrison 2014). Here, we use NH_4_^+^ to refer to ammonium + unionized ammonia (NH_4_^+^+NH_3_), both of which were present at the pH of the cultures (i.e., pH >8.0). We use the word “toxic” to describe concentrations of NH_4_^+^ that reduce phytoplankton growth by 50% or more, acknowledging that the majority of the toxic effect of NH_4_+NH_3_ may have been due to NH_3_ alone (e.g., Kalleqvist and Svenson 2007).

While changes in phytoplankton growth rate are typically used as the benchmark for interpreting toxicity effects, a more rapid response to NH_4_^+^ toxicity can be obtained by probing the quantum yield of photosystem II (PSII) in photosynthetic cells (Drath et al. 2008). PSII yield or efficiency, measured as variable over maximal fluorescence (F_v_/F_m_), is very sensitive to any condition that perturbs electron transport in the cell and is widely used in phytoplankton ecology to characterize stressful conditions for phytoplankton growth (Kromkamp and Forster 2003, Suggett et al. 2009), including nutrient limitation (Kolber et al. 1988, Geider et al. 1993, Kromkamp and Peene 1999, Berg et al. 2008), excessive irradiance or UV exposure (Behrenfeld et al. 1998, Six et al. 2007, Berg et al. 2011), oxidative stress (Drabkova et al. 2007), and toxicity from herbicides, pesticides, and other halogenated compounds (Muller et al. 2008, Choi et al. 2012, Kudela et al. 2015). The advantage of using F_v_/F_m_ is that the response time is on the order of minutes to hours following the onset of the stress, resulting in significant time savings compared with waiting for a response in growth rates (Kromkamp et al. 2005). In this study, we compared F_v_/F_m_, carbon assimilation, and growth in six species of phytoplankton to test their sensitivity to growth inhibition by NH_4_^+^, and to examine differences in growth rates according to inorganic N growth source.

## Materials and Methods

### Sampling locations and strain isolation

Near‐surface samples for phytoplankton isolations were collected using a plankton net at several stations in Suisun Bay and in the Sacramento River in the fall of 2013 and spring of 2014. Clonal cultures of six phytoplankton species, *Asterionella ralfsii*,* Fragilaria capucina*,* Thalassiosira weissflogii*,* Entomoneis paludosa*,* Chlorella minutissima*, and *Radiococcus planktonicus*, were established by micropipette isolations of single cells. *Asterionella ralfsii* and *F. capucina* were isolated from the Sacramento River (freshwater) while the other species were isolated from Suisun Bay (estuarine). The identity of the strains and purity of the cultures were confirmed by John Beaver (BSA Environmental) using microscopic evaluation and acid digestion of the diatom frustules. *Chlorella minutissima* and *R. planktonicus* are presently available from the National Center for Marine Algae and Microbiota under strain numbers CCMP3451 and CCMP3452, respectively. Strains were maintained in either filtered Sacramento River Water (SRW, salinity = 0) or filtered Monterey Bay seawater adjusted to a salinity of 10 with Millipore Milli‐Q water (MBSW, salinity = 10). Mixing with Milli‐Q water resulted in a dissolved inorganic carbon (DIC) concentration of ~700 μmol · L^−1^. Although lower than in Suisun Bay (i.e., Schemel 1984), the concentration was sufficient to maintain optimal growth as evidenced by the high F_v_/F_m_ in the cultures. Cultures were maintained on a 12:12 light:dark cycle under cool‐white fluorescent lights (85 μmol photons · m^−2^ · s^−1^ at the culture vessel surface) at a temperature of 15.5°C. These nutrient, temperature, and light conditions were comparable to those measured in Suisun Bay at the time of isolation of the cells.

### Experimental conditions

Stock cultures grown with NO_3_^−^ as the N‐source were transferred to media containing NH_4_^+^, at various concentrations, as the sole source of N for growth. After 1 week of growth, aliquots of the NH_4_^+^‐grown cells were concentrated by centrifugation and transferred into media containing NO_3_^−^, at various concentrations, as the sole source of N for growth. To start the experiment, cultures were spun down, rinsed with N‐free medium (salinity = 0 or 10), and re‐suspended in 200 mL medium in Erlenmeyer glass flasks containing SRW or MBSW with f/2 nutrient solution lacking N. To the MBSW base, silicate was added to a final concentration of f medium (i.e., twice the concentration of f/2 medium) to keep consistent concentrations between the SRW‐base (~200 μmoles silicate · L^−1^) and MBSW‐base media. To triplicate flasks, NH_4_^+^ was added to final concentrations of 20, 100, 200, 500, or 1,000 μmol · L^−1^ (low addition series), and 20, 100, 500, 1,000 or 3,000 μmol · L^−1^ (high addition series). The low and high addition series were used for strains with relatively lower and higher tolerance to NH_4_^+^, respectively. Relative tolerance levels were determined prior to the start of the experiments by simple growth tests using in vivo chlorophyll *a* (Chl *a*) fluorescence and F_v_/F_m_ as endpoints. After growth in NH_4_^+^‐medium for a week, aliquots of the cultures were spun down and re‐suspended in triplicate 200 mL Erlenmeyer flasks to which NO_3_^−^ was added to the same final concentrations as in the NH_4_^+^‐addition series. Because only the N concentration was varied among the treatments, and all other nutrients, trace metals and vitamins were kept constant, the nitrogen:phosphorus (N:P) ratio of the medium varied as follows: 20 μmol · L^−1^ (N:P = 1), 100 μmol · L^−1^ (N:P = 3), 200 μmol · L^−1^ (N:P = 6), 500 μmol · L^−1^ (N:P = 14), 1,000 μmol · L^−1^ (N:P = 28), 3,000 μmol · L^−1^ (N:P = 83). Culture biomass was inoculated at low levels and changes in F_v_/F_m_, cell abundance, Chl *a* and N concentrations were measured daily in order to characterize the growth response (Fig. S2 in the Supporting Information). Cultures were mixed by swirling prior to sampling each day.

### Measurements and sample analyses

The physiology of the strains was evaluated through a combination of measurements occurring either daily (F_v_/F_m_, Chl *a*, cell abundance) or once during mid‐exponential growth as for carbon (C) fixation.

The F_v_/F_m_ was measured by pulse‐amplitude‐modulated (PAM) fluorometry using a WATER‐PAM (Heinz‐Walz GmbH, Germany), with a standard array of three measuring light‐emitting diodes (LEDs) peaking in the red at 650 nm and 12 pulse LEDs peaking in the red at 660 nm. The WATER‐PAM was blanked with 0.2 μm filtered culture media. For measurements of F_v_/F_m_, aliquots were removed from the primary culture after swirling and dark adapted for 10 min. Potential biases caused by the short (10 min) dark‐adaptation period were checked by comparing F_v_/F_m_ values at 10, 20, 30, and 40 min from samples collected during exponential phase (concurrent with the carbon uptake experiments) for electron transport rate curves using the WATER‐PAM. There were no significant trends in dark‐adapted F_v_/F_m_ as a function of adaptation time. After dark adaptation, background Chl *a* fluorescence, F_0_, and maximal Chl *a* fluorescence following a saturating pulse (F_m_) was measured to derive the variable (F_v_) over maximum fluorescence according to:

(1) $$F_{v}/F_{m}=\left(F_{m}-F_{0}\right)/F_{m}$$

The percent suppression of F_v_/F_m_ over time in response to NH_4_^+^ was calculated as:

(2) $$\%\text{Suppression}=[1-\left(F_{v}/F_{m}\left(t\right)÷F_{v}/F_{m}\left(0\right))\right]\times 100$$

where F_v_/F_m_(0) is the initial F_v_/F_m_ at time zero and F_v_/F_m_(*t*) is the F_v_/F_m_ after exposure time *t*.

Samples for cell enumeration (all species except *Chlorella*) were preserved with acid Lugol's solution (20 μL Lugol's · mL^−1^ culture volume) and stored cool (4°C) until enumeration with a Zeiss (Thornwood, NY, USA) Axiovert 200 inverted microscope using a Parsons counting chamber. Abundances were estimated by random field counts totaling at least 400 unicells. Cell volumes were estimated by applying the geometric shapes that most closely matched the cell shape (Hillebrand et al. 1999). Volume calculations were based on measurements of the dimensions of 10 cells per strain. The abundance of *Chlorella* was measured by flow cytometry. Samples (3 mL) were fixed with 1% formaldehyde and analyzed using a Becton Dickinson Influx flow cytometer and cell sorter. Data acquisition was triggered on red fluorescence using stock cultures of *Chlorella* to set rejection gates for background noise. Samples were analyzed for 3–5 min and the number of events was normalized to volume counted to obtain cell abundance per unit volume. Samples for Chl *a* determination were collected onto uncombusted glass‐fiber filters (Whatman GF/F, Pittsburgh, PA, USA) and processed immediately using the non‐acidification method (Welschmeyer 1994). Samples for N (NO_3_^−^ and NH_4_^+^) analysis were filtered (Whatman GF/F) and stored frozen until processing. Ammonium was analyzed using the OPA method and relative fluorescence units were obtained via fluorometry (TD‐700; Turner Designs, San Jose, CA USA) according to Holmes et al. (1999). Nitrate was analyzed using a Lachat QuikChem 8500 Flow Injection Analyst System and Omnion 3.0 software (Lachat Instruments; Hach Company, Loveland, CO, USA). Nitrogen uptake rates were calculated from the ratio of the change in N concentration over time to the change in cell concentration over time to yield uptake as μmol N · cell^−1^.

Carbon uptake rates were measured as described in Kudela et al. (2006). Briefly, aliquots were removed from the cultures at noon and added to 25 mL glass scintillation vials to which ~1 μCi (~37,000 Bq) NaH^14^CO_3_ was added. The vials were subsequently incubated under the same light/temperature conditions as the cultures for ~60 min. ^14^C additions were calculated by measuring total activity using 1 mL volume from three random samples (per experiment), and time‐zero samples (three replicates) were collected by immediately spiking the vials with acid. Replicate samples for each light/nutrient treatment were inoculated and maintained in the dark to account for dark‐uptake. At the end of the incubation, the entire volume was acidified and allowed to degas for 24 h before 20 mL MP Biochemicals Ecolume scintillation cocktail was added. Samples were then counted using a Beckman 6500 liquid scintillation counter. Samples for DIC were filtered through GF/F filters and stored frozen until analysis. DIC concentration in the samples was measured on a Shimadzu (Columbia, MD, USA) total carbon/total nitrogen system according to manufacturer's directions. We did not have samples available from all experiments therefore a subset of samples was analyzed from each set of experiments. Measured DIC concentrations varied by less than 10% across treatments. The lowest DIC was in the high‐biomass treatments, but no measured DIC was less than 600 μmol · L^−1^ suggesting that carbon‐limitation was not a significant issue. Biomass‐dependent correction factors for DIC consumption were calculated for each experiment based on the measured DIC concentrations. These were used to estimate final DIC concentrations in each culture. Carbon uptake rates were calculated from scintillation counts and final DIC concentrations after adjusting for the time‐zero blank and correcting for dark‐uptake. Carbon assimilation rates were obtained by normalizing C uptake rates to Chl *a* (mg C · mg Chl^−1^ · h^−1^), hereafter referred to simply as “C assimilation.”

To directly assess the impact of transient additions of either NH_4_^+^ or NO_3_^−^ on productivity in the cultures, samples from cultures grown on 20 μmol · L^−1^ NO_3_^−^ collected during mid‐exponential growth were split into two aliquots that were incubated for 24 h following an addition of either 5 μmol NO_3_^−^ · L^−1^ or 5 μmol NH_4_^+^ · L^−1^. At the end of the incubation, C fixation was measured by adding ^14^C‐labeled bicarbonate and incubating for an additional h using the same environmental conditions.

The rate of cell‐specific growth on each N source was computed by fitting the exponential function to the data:

(3) $$C=C_{0}e^{kt}$$

Where *C* is the cell abundance, *C*_0_ is the starting cell abundance, *k* is the growth constant (d^−1^), and *t* is time. Two‐way analysis of variances (ANOVAs) were conducted on all the data using species and N source as factors; in tests with significant interactions, two‐way ANOVAs were also conducted within each species using N source and concentration as factors. All calculations and statistical tests were carried out using R software (R Core Team 2016).

## Results

### Species‐specific differences in physiological responses

Two‐way ANOVAs were performed to determine whether there was an effect related to N source or species on the mean response of a range of physiological parameters. With respect to most, there was a significant effect of species but not of N source (Table 1).

**Table 1:** Probabilities and F values (in parenthesis) resulting from two‐way ANOVAs of F_v_/F_m_, C‐assimilation (mg C · mg Chl^−1^ · h^−1^), growth rate (d^−1^), Chl *a* (pg per cell), and N uptake (μmol N per cell) using species and N source as factors. Significant probabilities (α  ≤  0.05) in bold

Parameter	Species (factor 1), df = 5	N Source (factor 2), df = 1	Interaction, df = 5	Residuals
F_v_/F_m_	**<2.2 × 10** ^**−16**^ **(83)**	0.051 (3.9)	**7.88 × 10** ^**−9**^ **(11)**	df = 114
C assimilation	**7.2 × 10** ^**−6**^ **(15)**	**9.1 × 10** ^**−6**^ **(37)**	**1.6 × 10** ^**−4**^ **(9.3)**	df = 78
Growth Rate	**4.5 × 10** ^**−10**^ **(13)**	0.120 (2.5)	**7.2 × 10** ^**−9**^ **(11)**	df = 114
Chl *a*	**<2.2 × 10** ^**−16**^ **(936)**	0.450 (0.55)	0.051 (3.3)	df = 880
N uptake	**6.8 × 10** ^**−6**^ **(24)**	0.850 (0.04)	0.600 (0.75)	df = 12

**Figure 1:**
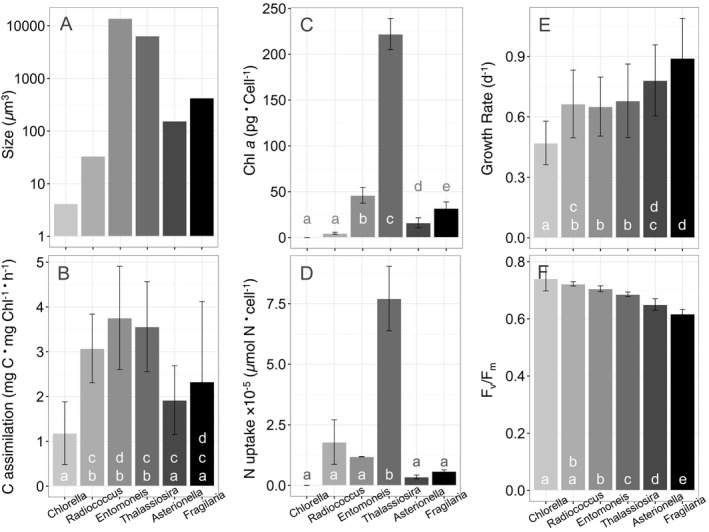
Species‐specific differences in (A) size (μm^3^), (B) Carbon assimilation (mg C · mg Chl^−1^ · h^−1^), (C) Chl a per cell, (D) Nitrogen uptake (μmol N per cell), (E) Cell‐specific growth rate (d^−1^), and (F) F_v_/F_m_. Treatment means were compared using a two‐way ANOVA (with species and nutrient source as the factors) and a pair‐wise means comparison test. Means that are not significantly different are labeled with the same letter (α = 0.05).

The phytoplankton strains differed by three orders of magnitude in average cell volume (Fig. 1a). The smallest species were the chlorophytes *C. minutissima* and *R. planktonicus*, 4 and 33 μm^3^, respectively, and the largest species were the diatoms *T. weissflogii* and *E. paludosa*, 6,430 and 13,850 μm^3^, respectively. The chain‐forming freshwater diatoms *A. ralfsii* and *F. capucina* were intermediate in average cell volume at 155 and 427 μm^3^, respectively (Fig. 1a). Relative differences in C assimilation were similar to relative differences in size among species, with *E. paludosa* and *T. weissflogii* having the greatest rates of C assimilation (Fig. 1, a and b). Chl *a* per cell was significantly greater in *T. weissflogii* compared with any other species whereas *C. minutissima* had the least amount of Chl *a* per cell (Fig. 1c). Nitrogen uptake per cell was also significantly greater in *T. weissflogii* compared with the other species (Fig. 1d). Again, N uptake per cell was least for *C. minutissima* (Fig. 1d). The fastest mean cell‐specific growth rates were observed in *F. capucina* (0.89 ± 0.19 · d^−1^) and *A. ralfsii* (0.78 ± 0.17 · d^−1^) while *C. minutissima* grew significantly slower (0.47 ± 0.10 · d^−1^) than the other isolated genera (Fig. 1e). Relative differences in mean cell‐specific growth rates among species did not correspond with relative differences in carbon assimilation and N uptake rates in that the fastest growing species, *F. capucina* and *A. ralfsii*, had the second to lowest rates of C assimilation and N uptake (Fig. 1, b, d, and e). At concentrations of nutrients that were not toxic, maximal F_v_/F_m_ was 0.6 or above in all the cultures (Fig. 1f).

### Effect of N source and concentration on productivity and growth

Although N source in most cases did not have a significant effect on the mean response of most physiological parameters, it did exhibit a significant effect in the mean response of C assimilation (Table 1). However, growth rate, C assimilation and F_v_/F_m_ all exhibited significant interactions of species with N source, such that the effect of the N source varied depending on species (Table 2). Analyzing the variance of both N type and concentration within each species at the concentration range where NH_4_^+^ did not appear to be toxic demonstrated significant effect of N type in some species and not in others (Table 2).

**Table 2:** Probabilities and *F*‐values (in parenthesis) resulting from within‐species two‐way ANOVAs of F_v_/F_m_, C‐assimilation, and growth rate using N source and concentration as factors. Significant probabilities (α  ≤  0.05) in bold

Species	df	Factor	F_v_/F_m_	Carbon‐assimilation	Growth rate
*Chlorella* a	1	N type	**2.26 × 10** ^**−7**^ **(51)**	0.051 (3.7)	**1.30 × 10** ^**−14**^ **(273)**
	5	Concentration	**2.06 × 10** ^**−3**^ **(5.3)**	0.094 (2.1)	**1.13 × 10** ^**−6**^ **(15)**
	5	Interaction	**7.20 × 10** ^**−3**^ **(6.3)**	0.850 (0.4)	0.340 (1.2)
	24	Residuals			
*Radiococcus* a	1	N type	**3.23 × 10** ^**−7**^ **(56)**	**7.4 × 10** ^**−6**^ **(42)**	**1.08 × 10** ^**−6**^ **(47)**
	4	Concentration	0.320 (1.3)	0.052 (3.7)	0.080 (2.5)
	4	Interaction	0.350 (1.2)	0.390 (1.0)	0.056 (3.2)
	20	Residuals			
*Entomoneis* b	1	N type	0.130 (2.6)	0.290 (1.2)	**2.90 × 10** ^**−3**^ **(12)**
	3	Concentration	0.720 (0.3)	0.062 (3.7)	0.350 (1.2)
	3	Interaction	0.230 (1.7)	0.058 (3.4)	0.063 (3.8)
	16	Residuals			
*Thalassiosira* b	1	N type	**3.21 × 10** ^**−6**^ **(41)**	0.290 (1.2)	**1.2 × 10** ^**−11**^ **(288)**
	3	Concentration	0.053 (3.2)	0.062 (2.9)	0.052 (3.1)
	3	Interaction	0.051 (3.1)	0.058 (3.0)	0.220 (1.6)
	16	Residuals			
*Asterionella* c	1	N type	0.930 (0.0)	**3.6 × 10** ^**−9**^ **(228)**	0.450 (0.6)
	2	Concentration	0.360 (1.1)	0.064 (3.1)	0.072 (3.3)
	2	Interaction	0.510 (0.7)	0.062 (3.2)	0.052 (4.2)
	12	Residuals			
*Fragilaria* c	1	N type	0.170 (1.9)	**1.4 × 10** ^**−8**^ **(526)**	0.390 (0.8)
	2	Concentration	0.100 (2.4)	0.058 (4.5)	0.430 (0.9)
	2	Interaction	0.850 (0.2)	0.310 (1.1)	0.640 (0.5)
	12	Residuals			

a 3,000 μmoles N · L^−1^ and below.

b 1,000 μmoles N · L^−1^ and below.

c 200 μmoles N · L^−1^ and below.

**Figure 2:**
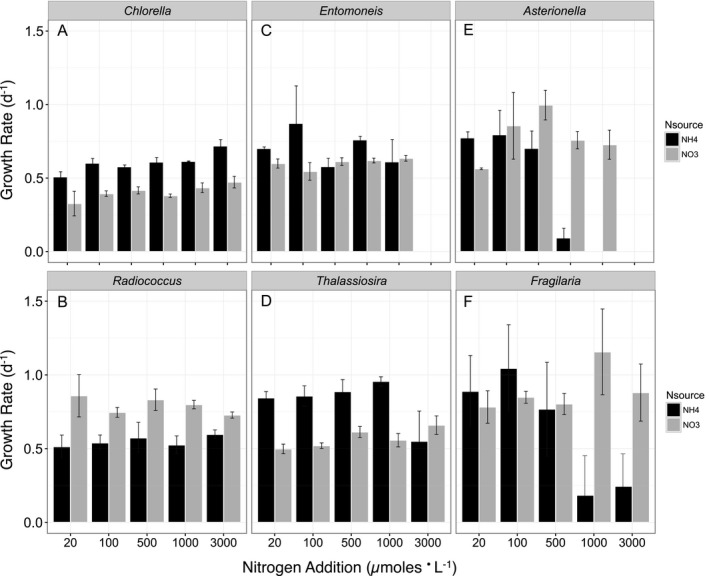
Cell‐specific growth Rates (d^−1^) as a function of N concentration and N source for the chlorophytes (A) *Chlorella minutissima*, (B) *Radiococcus planktonicus*, and the estuarine diatoms (C) *Entomoneis paludosa*, (D) *Thalassiosira weissflogii*, and the freshwater diatoms (E) *Asterionella ralfsii* and (F) *Fragilaria capucina*. Each bar (black=NH_4_^+^ as the N source, grey=NO_3_^−^ as the N source) represents the mean and standard deviation of triplicate cultures. The rate of growth on each N source was computed by fitting the exponential function *C=C*_0_ *e* ^*kt*^ to the data where *C* is the cell abundance, *C*_0_ is the starting cell abundance, *k* is the growth constant (d^−1^), and *t* is time.

With the exception of *R. planktonicus*, rates of growth (estimated from changes in cell abundance) were generally faster when growing on NH_4_^+^ compared with NO_3_^−^ as a sole source of N (Fig. 2, a–f). At a concentration of 1,000 μmoles · L^−1^ or below, cell‐specific growth rates of *T. weissflogii*,* C. minutissima* and *E. paludosa* were 61%, 49% and 20%, respectively, greater on NH_4_^+^ than NO_3_^−^ (Fig. 2, a, c and d). These differences were significant for all three species (Table 2). At a concentration of 100 μmoles · L^−1^ and below, growth rates of *F. capucina* and *A. ralfsii* were 18% and 10% greater on NH_4_^+^ than NO_3_^−^ (Fig. 2, e and f). These differences in the growth rates with N type were not significant (Table 2). *Radiococcus planktonicus* had a 35% lower growth rate on NH_4_^+^ than NO_3_^−^ (Fig. 2b) which was significant (Table 2).

**Figure 3:**
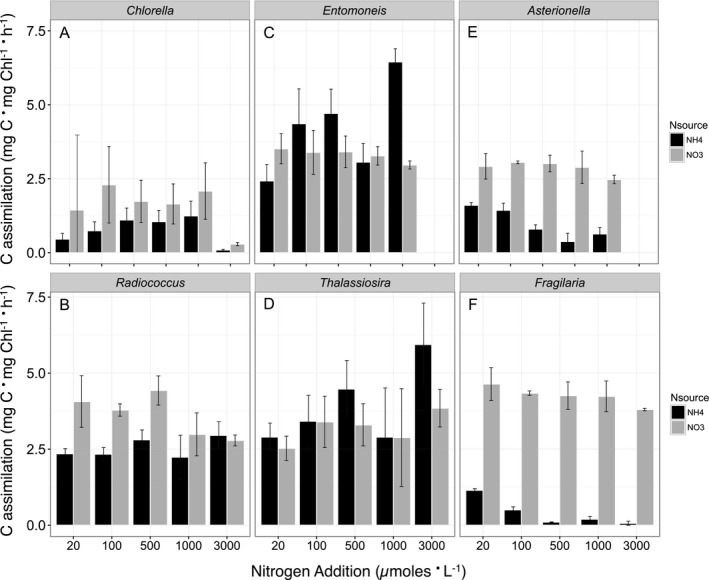
Carbon assimilation (mg C · mg Chl^−1^ · h^−1^) in mid‐exponential phase as a function of N concentration and N source for the chlorophytes (A) *Chlorella minutissima*, (B) *Radiococcus planktonicus*, and the estuarine diatoms (C) *Entomoneis paludosa*, (D) *Thalassiosira weissflogii*, and the freshwater diatoms (E) *Asterionella ralfsii* and (F) *Fragilaria capucina*. Each bar (black=NH_4_^+^ as the N source, grey=NO_3_^−^ as the N source) represents the mean and standard deviation of triplicate cultures.

In contrast with rates of cell‐specific growth, four out of the six species exhibited higher rates of C assimilation when growing on NO_3_^−^ compared with NH_4_^+^ (Fig. 3, a–f). Both *C. minutissima* and *R. planktonicus* exhibited greater rates of C assimilation when growing on NO_3_^−^ than NH_4_^+^ below 3,000 μmoles · L^−1^, but the difference was only significant in *R. planktonicus* (Table 2; Fig. 3, a and b). *Entomoneis paludosa* and *T. weissflogii* exhibited no significant difference in C assimilation with N source below 1,000 μmol · L^−1^ (Fig. 3, c and d). In contrast, rates of C assimilation were significantly greater when growing on NO_3_^−^ than on NH_4_^+^ in *F. capucina* and *A. ralfsii* (Table 2) at concentrations below 500 μmol · L^−1^ (Fig. 3, e and f). For example, at 20 μmol · L^−1^ N, C assimilation was 4‐ and 2‐fold greater on NO_3_^−^ relative to NH_4_^+^, for *F. capucina* and *A. ralfsii*, respectively (Fig. 3, e and f).

**Figure 4:**
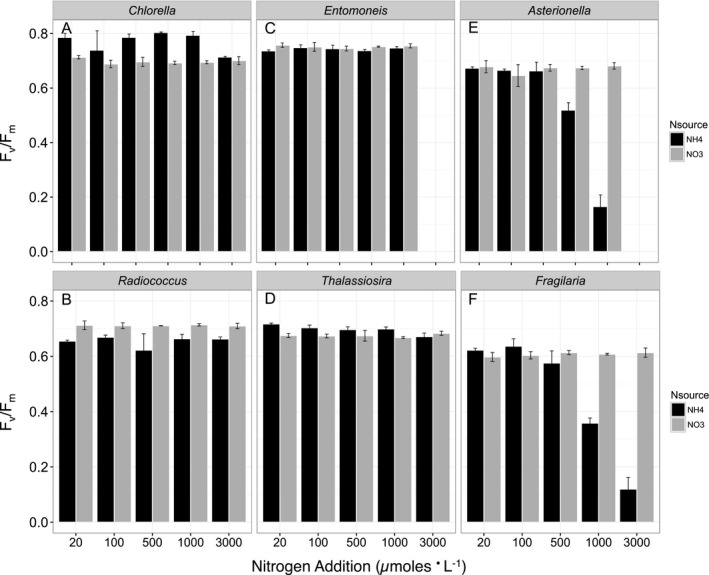
Phytoplankton F_v_/F_m_ in mid‐exponential phase as a function of N concentration and N source for the chlorophytes (A) *Chlorella minutissima*, (B) *Radiococcus planktonicus*, and the estuarine diatoms (C) *Entomoneis paludosa*, (D) *Thalassiosira weissflogii*, and the freshwater diatoms (E) *Asterionella ralfsii* and (F) *Fragilaria capucina*. Each bar (black=NH_4_^+^ as the N source, grey=NO_3_^−^ as the N source) represents the mean and standard deviation of triplicate cultures.

Patterns in F_v_/F_m_ with N source mirrored patterns in growth rates with N source (Figs. 2 and 4). In *C. minutissima*, F_v_/F_m_ was significantly greater when growing on NH_4_^+^ than when growing on NO_3_^−^. In contrast, F_v_/F_m_ in *R. planktonicus* was significantly greater when growing on NO_3_^−^ compared with NH_4_^+^ (Fig. 4, a and b). At 1,000 μmol · N L^−1^ and below, there was no difference in F_v_/F_m_ with N source in *E. paludosa* (Fig. 4c), but F_v_/F_m_ was significantly greater in *T. weissflogii* when growing on NH_4_^+^ than when growing on NO_3_^−^ (Fig. 4d). Below 200 μmol · L^−1^, there was no impact of N source on F_v_/F_m_ in *A. ralfsii* or *F. capucina*. Above 200 μmol · L^−1^, there was a significant negative effect of NH_4_^+^ concentration on F_v_/F_m_ (*F*_1,7_ = 255, *P* = 9.2 × 10^−7^ for *A. ralfsii* and *F*_1,7_ = 54, *P* = 1.5 × 10^−4^ for *F. capucina*) in both species (Fig. 4, e and f).

### Toxicity effects

**Figure 5:**
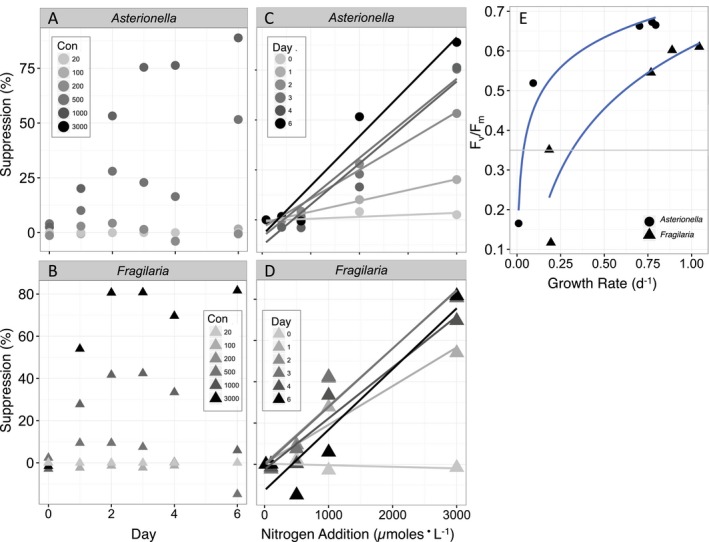
Percent suppression of F_v_/F_m_ as a function of day with increasing additions of NH_4_^+^ (20–3,000 μmoles · L^−1^) for (A) *Asterionella ralfsii*, and (B) *Fragilaria capucina*. Percent suppression of F_v_/F_m_ as a function of NH_4_^+^ concentration at different days for (C) *Asterionella ralfsii*, and (D) *Fragilaria capucina*. (E) Changes in F_v_/F_m_ as a function of cell‐specific growth rate (d^−1^) for *A. ralfsii* (solid circles; F_v_/F_m_ = 0.109 ln (Growth Rate)+0.71, *r* ^2^ = 0.97, *P* = 0.0026) and *F. capucina* (solid triangles, F_v_/F_m_ = 0.224 ln (Growth Rate)+0.61, *r* ^2^ = 0.83, *P* = 0.032). [Color figure can be viewed at wileyonlinelibrary.com]

Based on this six‐species comparison, *A. ralfsii* and *F. capucina* were the most sensitive to NH_4_^+^ toxicity as evidenced by suppression in F_v_/F_m_, C assimilation, and growth, at higher concentrations of NH_4_^+^ (Figs. 2, 3, 4). Suppression of F_v_/F_m_ was evident after 1 h (data not shown) and significant after only 1 day in both species (Fig. 5, a and b). In *A. ralfsii*, suppression continued to increase linearly at the highest NH_4_^+^ concentration with each day, whereas in *F. capucina* suppression increased until day 2 then leveled off (Fig. 5, a and b). Suppression was approximately linear as a function of NH_4_^+^ concentration regardless of the day (Fig. 5, c and d). For *A. ralfsii*, the degree of suppression increased each day such that the steepest slope was observed on day 6 when >75% suppression occurred at the highest NH_4_^+^ concentration. For *F. capucina*, the maximum degree of suppression was reached on day 2 (Fig. 5, c and d). Although suppression of F_v_/F_m_ was linear with NH_4_^+^ concentration above a concentration of 200 μmoles · L^−1^, decrease in growth rate was not and F_v_/F_m_ declined logarithmically as a function of growth rate decreases (Fig. 5e). Below F_v_/F_m_ of 0.35, growth rates did not decrease further in either *A. ralfsii* or *F. capucina*. These data suggest that an F_v_/F_m_ of 0.35 represents the point where minimal growth rates were reached (Fig. 5e).

The concentration of NH_4_^+^ at which cell‐specific growth was depressed by 50% occurred at ~350 μmoles NH_4_^+^ · L^−1^ and ~800 μmoles NH_4_^+^ · L^−1^, for *A. ralfsii* and *F. capucina*, respectively (Table S1 in the Supporting Information). *Asterionella ralfsii* was acutely sensitive at concentrations above 200 μmoles NH_4_^+^ · L^−1^ with an 88% decrease in growth rate at a concentration of 500 μmoles NH_4_^+^ · L^−1^. *Fragilaria capucina* was not as sensitive to NH_4_^+^ toxicity exhibiting only a 14% decrease in growth rate at 500 μmoles NH_4_^+^ · L^−1^ but an 80% decrease at 1,000 μmoles NH_4_^+^ · L^−1^. *Entomoneis paludosa* and *T. weissflogii* were not sensitive to NH_4_^+^ concentration at 1,000 μmoles NH_4_^+^ · L^−1^ or below. There was a 48% decrease in growth rate between 1,000 and 3,000 μmoles NH_4_^+^ · L^−1^ in *T. weissflogii*, with corresponding decreases in F_v_/F_m_ and C assimilation (Figs. 2, 3, 4; Table S1). The chlorophytes *C. minutissima* and *R. planktonicus* were the two most tolerant strains to NH_4_^+^ with a toxicity threshold ~3,000 μmoles NH_4_^+^ · L^−1^. Growth rates of *C. minutissima* were invariant with increases in NH_4_^+^ concentration between 100 and 1,000 μmoles NH_4_^+^ · L^−1^, but increased 21% between 1,000 and 3,000 μmoles NH_4_^+^ · L^−1^. Growth rates of *R. planktonicus* increased 40% between 100 and 1,000 μmoles NH_4_^+^ · L^−1^, then decreased 30% between 1,000 and 3,000 μmoles · L^−1^; however, at 3,000 μmoles NH_4_^+^ · L^−1^ rates were still 16% above those measured at 20 μmoles NH_4_^+^ · L^−1^ (Fig. 2; Table S1).

With the exception of *C. minutissima*, which evidenced an increase in the rate of growth at 3,000 μmoles N · L^−1^ and a dissolved N:P ratio of 83, changes in the dissolved N:P ratio of the medium had no impact on C assimilation or growth rates in any of the species tested here below their toxicity thresholds (Table S2 in the Supporting Information). This was consistent with the effect of changes in N concentration (Table 2), demonstrating a lack of effect of changes in dissolved nutrient ratios, from 1 to 83, at non‐limiting nutrient concentrations.

### Effect of small, transient pulses of N on productivity

**Figure 6:**
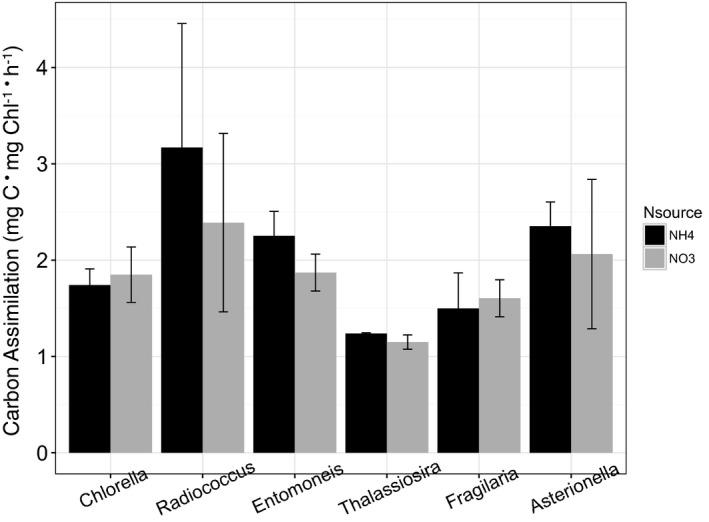
Carbon assimilation (mg C · mg Chl^−1^ · h^−1^) 24 h after exposure to either 5 μmoles NO_3_^−^ · L^−1^ or 5 μmoles NH_4_^+^ · L^−1^, in cultures growing on 20 μmoles NO_3_^−^ · L^−1^.

To test whether low additions of NH_4_^+^ would decrease productivity in cells growing on NO_3_^−^, the effect of adding 5 μmoles NH_4_^+^ · L^−1^ on C assimilation was compared with the effect of adding 5 μmoles NO_3_^−^ · L^−1^. The effect of NH_4_^+^ addition was either no different than that of NO_3_^−^ addition, or it stimulated productivity. The former was true for *C. minutissima*,* T. weissflogii*, and *F. capucina*, whereas the latter was true for *R. planktonicus*,* E. paludosa*, and *A. ralfsii* (Fig. 6). Productivity was stimulated 32% by a transient addition of NH_4_^+^ compared to addition of NO_3_^−^ in *R. planktonicus*; this was the largest difference among the six species assayed (Fig. 6).

## Discussion

### NH_4_^+^ toxicity thresholds

The results from testing four species of diatoms and two species of chlorophytes exposed to a range of NH_4_^+^ concentrations demonstrated that only two of the species, *A. ralfsii* and *F. capucina*, exhibited toxicity effects at the concentrations of NH_4_^+^ tested here, and, that these effects were not evident below a concentration of 200 μmoles NH_4_^+^ · L^−1^. This threshold was corroborated by three different endpoints including F_v_/F_m_, carbon assimilation, and growth rate. As a consequence, it does not appear that toxicity to NH_4_^+^ provides a physiological explanation for why diatoms would potentially grow more slowly when exclusively using NH_4_^+^ compared with NO_3_^−^ at environmental concentrations of NH_4_^+^.

Above a concentration of 200 μmoles NH_4_^+^ · L^−1^, changes in F_v_/F_m_ provided a rapid and reliable method of detecting the NH_4_^+^ toxicity response. Toxicity to NH_4_^+^ was manifested by a suppression of F_v_/F_m_ in a dose‐dependent manner that was significant after 1 day, providing a substantial time savings over traditional 4‐day growth bioassays to detect toxicity. In *A. ralfsii* and *F. capucina*, F_v_/F_m_ displayed a logarithmic relationship with growth rates, where minimal growth rates were reached at an F_v_/F_m_ of 0.35. Below this threshold, growth rates did not decrease further but F_v_/F_m_ rapidly decreased to near‐zero suggesting that an F_v_/F_m_ of ~0.35 represented a point of “no return” for phytoplankton growth in the two cultures examined here. However, because F_v_/F_m_ cannot be compared in an absolute sense among species (or taxonomic groups) as F_0_ may vary as a function of the accessory pigments or ratios of photosystems I and II (Schreiber 2004, Suggett et al. 2009), this threshold may not hold for other species of phytoplankton.

Recent studies suggest that the effect of NH_4_^+^ toxicity in phytoplankton is actually due to unionized NH_3_ which competitively binds with the oxygen evolution complex, inhibits the water splitting reaction, and causes direct damage to the PSII reaction center protein D1 (Kallqvist and Svenson 2003, Drath et al. 2008). Damage to PSII from NH_3_ is accelerated in mutants lacking D1 protein repair enzymes, as well as under high light (Drath et al. 2008). In contrast with NH_4_^+^, whose transport across the plasma membrane is tightly regulated by the transporter AMT1, NH_3_ can diffuse freely into the cell (Loque et al. 2009). The fraction of total ammonia (NH_4_^+^ + NH_3_) that is comprised of NH_3_ varies depending on temperature and pH, and increases substantially above pH 9.2 (Khoo et al. 1977). At a given temperature and pH, the amount of NH_3_ increases with increased NH_4_^+^ concentration; therefore F_v_/F_m_ suppression and growth inhibition increases in a dose‐dependent manner with NH_4_^+^ concentration (Kallqvist and Svenson 2003, Drath et al. 2008). Based on our experiments it's clear that lower growth rates observed in the chlorophyte *R. planktonicus* on NH_4_^+^ compared with NO_3_^−^ were not due to NH_4_^+^ toxicity as the difference in the growth rate between NH_4_^+^ and NO_3_^−^ did not increase with increasing concentrations of NH_4_^+^.

Given that 3%–6% of NH_4_^+^ is unionized NH_3_ at a salinity of 10, temperature of 15°C, and pH of 8.0–8.3 (i.e., Khoo et al. 1977), we calculate that *A. ralfsii* has a toxicity threshold of ~15–20 μmoles NH_3_ · L^−1^ and *F. capucina* has a toxicity threshold of 30–44 μmoles NH_3_ · L^−1^. Because we isolated all the species in these experiments at the same time, and cultured them under the same conditions, it is clear that the differences in the NH_4_^+^ toxicity thresholds among them is due to inherent genetic differences, for example, in accordance with the efficiency of their D1 protein repair cycles, and not due to an acclimation response. Moreover, the toxicity thresholds differed according to taxa and agree with previously published thresholds in that chlorophytes are substantially more resistant to NH_4_^+^ toxicity than diatoms, although diatom thresholds vary widely (Collos and Harrison 2014 and references therein). In turn, diatoms appear more resistant to NH_4_^+^ toxicity than dinoflagellates and some raphidophytes that have relatively low NH_4_^+^ tolerance thresholds (Clark and Flynn 2002, Suksomjit et al. 2009, Collos and Harrison 2014).

### Differences in growth rates on NH_4_^+^ and NO_3_^−^

While toxicity thresholds appear to vary according to taxa, differences in growth rates on NH_4_^+^ and NO_3_^−^ do not. Under the conditions in this study, the diatom *T. weissflogii* and the chlorophyte *C. minutissima* both grew nearly 50% faster on NH_4_^+^ compared with NO_3_^−^. The only isolate that demonstrated a significantly faster rate of growth on NO_3_^−^ compared with NH_4_^+^ was the chlorophyte *R. planktonicus*. Comparing the results obtained in this study with a number of similar culture investigations illustrates that variation in growth rates with NH_4_^+^ and NO_3_^−^ is highly species‐specific (Table 3). Therefore, the notion that diatoms as a group grow better on NO_3_^−^ and members of other phytoplankton groups grow better on NH_4_^+^ is not borne out in these culture studies. It appears that most phytoplankton, including diatoms, grow faster when using NH_4_^+^ compared with NO_3_^−^ as a sole source of N for growth, but that this difference is typically on the order of ≤25% (Table 3).

**Table 3:** Percent difference in growth rate, μ, of phytoplankton growing on NH_4_^+^ vs. NO_3_^−^ [(μ_NH4_/μ_NO3−1_) × 100] as the sole source of N for growth

Taxon	Species	Difference (%)	Source	Culture conditions
Diatom	*Thalassiosira weissflogii*	61.9	This study	Batch culture, 16°C, 85 μmol photons · m^−2^ · s^−1^ (L:D cycle)
Chlorophyte	*Chlorella minutissima*	49.7	This study	Batch culture, 16°C, 85 μmol photons · m^−2^ · s^−1^ (L:D cycle)
Diatom	*Thalassiosira pseudonana*	39.0	Clark and Flynn 2000	Batch culture, 16°C, 200 μmol photons · m^−2^ · s^−1^ (L:D cycle)
Raphidophyte	*Heterosigma carterae* a	31.0	Wood and Flynn 1995	Batch culture, 18°C, 50, 200, 350 μmol photons · m^−2^ · s^−1^ (L:D cycle)
Raphidophyte	*Heterosigma carterae*	29.3	Clark and Flynn 2000	Batch culture, 16°C, 200 μmol photons · m^−2^ · s^−1^ (L:D cycle)
Diatom	*Pseudo‐nitzschia calliantha* b	24.4	Thessen et al. 2009	Batch culture, 15°C, 150–200 μmol photons · m^−2^ · s^−1^ (L:D cycle)
Diatom	*Skeletonema costatum*	21.1	Tada et al. 2009	Batch culture, 21°C, 30°C, 150 μmol photons · m^−2^ · s^−1^ (L:D cycle)
Cyanobacterium	*Cylindrospermopsis raciborskii* b	20.7	Saker and Neilan 2001	Batch culture, 25°C, 50 μmol photons · m^−2^ · s^−1^ (L:D cycle)
Diatom	*Entomoneis paludosa*	20.3	This study	Batch culture, 16°C, 85 μmol photons · m^−2^ · s^−1^ (L:D cycle)
Dinoflagellate	*Prorocentrum minimum*	19.2	Fan et al. 2003	Semi‐batch culture, 20°C, 100 μmol photons · m^−2^ · s^−1^ (L:D cycle)
Diatom	*Fragilaria capucina*	18.5	This study	Batch culture, 16°C, 85 μmol photons · m^−2^ · s^−1^ (L:D cycle)
Chlorophyte	*Dunaliella tertiolecta* a	18.1	Paasche 1971	Batch culture, 25°C, 55, 300 μmol photons · m^−2^ · s^−1^ (L:D cycle)
Haptophyte	*Emiliania huxleyi*	15.4	Strom and Bright 2009	Batch culture, 15°C, 150–200 μmol photons · m^−2^ · s^−1^ (L:D cycle)
Diatom	*Pseudo‐nitzschia fraudulenta* b	14.8	Thessen et al. 2009	Batch culture, 15°C, 150–200 μmol photons · m^−2^ · s^−1^ (L:D cycle)
Diatom	*Thalassiosira pseudonana*	13.8	Levasseur et al. 1993	Batch culture, 18°C, 170 μmol photons · m^−2^ · s^−1^ (Continuous light)
Diatom	*Asterionella ralfsii*	10.5	This study	Batch culture, 16°C, 85 μmol photons · m^−2^ · s^−1^ (L:D cycle)
Raphidophyte	*Heterosigma akashiwo*	8.5	Herndon and Cochlan 2007	Batch culture, 15°C, 110 μmol photons · m^−2^ · s^−1^ (Continuous light)
Diatom	*Thalassiosira weissflogii* c	8.5	Clark and Flynn 2000	Batch culture, 16°C, 200 μmol photons · m^−2^ · s^−1^ (L:D cycle)
Chlorophyte	*Stichococcus bacillaris* c	5.0	Clark and Flynn 2000	Batch culture, 16°C, 200 μmol photons · m^−2^ · s^−1^ (L:D cycle)
Dinoflagellate	*Gymnodinium sanguineum*	4.9	Levasseur et al. 1993	Batch culture, 18°C, 170 μmol photons · m^−2^ · s^−1^ (Continuous light)
Diatom	*Thalassiosira pseudonana* a	4.9	Parker and Ambrust 2005	Semi‐Batch culture, 22°C, 50, 300 μmol photons · m^−2^ · s^−1^ (Continuous light)
Chlorophyte	*Dunaliella tertiolecta*	0.7	Levasseur et al. 1993	Batch culture, 18°C, 170 μmol photons · m^−2^ · s^−1^ (Continuous light)
Diatom	*Pseudo‐nitzschia multiseries* b	−2.8	Thessen et al. 2009	Batch culture, 15°C, 150–200 μmol photons · m^−2^ · s^−1^ (L:D cycle)
Haptophyte	*Emiliania huxleyi* c	−4.0	Clark and Flynn 2000	Batch culture, 16°C, 200 μmol photons · m^−2^ · s^−1^ (L:D cycle)
Pelagophyte	*Aureococcus anophagefferens*	−5.4	Berg et al. 2008	Batch culture, 18°C, 45 μmol photons · m^−2^ · s^−1^ (L:D cycle)
Diatom	*Thalassiosira weissflogii*	−14.7	Fan et al. 2003	Semi‐Batch culture, 20°C, 100 μmol photons · m^−2^ · s^−1^ (L:D cycle)
Diatom	*Chaetoceros gracilis*	−15.2	Levasseur et al. 1993	Batch culture, 18°C, 170 μmol photons · m^−2^ · s^−1^ (Continuous light)
Chlorophyte	*Radiococcus planktonicus*	−25.0	This study	Batch culture, 16°C, 85 μmol photons · m^−2^ · s^−1^ (L:D cycle)
All	Mean	13.3 ± 19		

a Percent difference calculated from two or more irradiance levels.

b Percent difference calculated based on a mix of strains of the same species.

c Percent difference calculated from carbon‐specific growth rates, C_μ_, at DIC ≥1 mM.

Although we did not test different strains of the same species in this study, others have found that differences in growth rate when using NH_4_^+^ compared to using NO_3_^−^ varies as much among strains within a species as among different species (Saker and Neilan 2001, Thessen et al. 2009). For example, eight strains of the harmful cyanobacteria *Cylindrospermopsis raciborskii* grew on average 20% faster on NH_4_^+^ than they did on NO_3_^−^, but ranged from −33% to 103% depending on the strain (Saker and Neilan 2001). Similarly, percent differences in growth on NH_4_^+^ compared with NO_3_^−^ in five strains of the diatom *Pseudo‐nitzschia fraudulenta* ranged from −17% to 67%, with an average of 15% faster growth on NH_4_^+^ compared with NO_3_^−^ (Thessen et al. 2009). Based on these data one cannot conclude that cyanobacteria, or chlorophytes, are at an advantage when growing on NH_4_^+^ because diatoms have the same advantage.

As a group, the diatoms in this study exhibited faster rates of growth compared to the chlorophytes. This difference in growth rates among taxonomic groups coupled with initial phytoplankton community composition may matter more for final phytoplankton community composition than initial composition of the N pool. This is difficult to test under natural conditions because NH_4_^+^ very rarely dominates the total N pool in marine systems. But, a few investigations from eutrophic coastal communities demonstrate that when that is the case, and diatoms are present in the initial assemblage, they outcompete other phytoplankton and form monospecific blooms (Admiraal 1977, Tada et al. 2001, Esparza et al. 2014). For example, blooms of the diatom *Skeletonema* sp. dominates eutrophic Dokai Bay, Japan, where NH_4_^+^ concentrations are typically >200 μmoles · L^−1^ (Suksomjit et al. 2009, Tada et al. 2009). Similarly, NH_4_^+^ is the main source of N sustaining summer blooms of the diatoms *Skeletonema costatum*,* Thalassiosira* spp. and *Chaetoceros* spp. in Hong Kong coastal waters (Xu et al. 2009, 2012). Therefore, patterns observed in field investigations linking diatoms to NO_3_^−^ uptake are probably due to NH_4_^+^ being depleted more quickly, leaving only NO_3_^−^ at a high enough concentration at the time that diatom biomass starts to accumulate in the early stages of a bloom, and not because diatoms prefer NO_3_^−^ or grow faster on NO_3_^−^ than NH_4_^+^.

An interesting question is why would different phytoplankton species have evolved to grow at slightly different rates when using NH_4_^+^ vs. NO_3_^−^ as a sole source of N for growth? A common argument is that it is energetically more favorable for phytoplankton to grow on NH_4_^+^ compared with NO_3_^−^ because reductant does not need to be expended to reduce NO_3_^−^ to NH_4_^+^ before the N can be assimilated, saving the cell greater than 20% on energy costs (Syrett 1981, Thompson et al. 1989, Levasseur et al. 1993). This extra cost may be reflected in a greater photosynthetic quotient (mol O_2_ evolved per CO_2_ assimilated) or Chl *a* per cell (Raine 1983, Thompson et al. 1989). In addition to the energetic expenditure, reduction in NO_3_^−^ to NH_4_^+^ requires the processing of the N through an extra enzyme pathway, which at higher growth rates can lead to an enzymatic bottleneck. In turn, the bottleneck may result in lower N and protein contents, leading cells grown on NO_3_^−^ to appear more N stressed (Wood and Flynn 1995, Page et al. 1999) and exhibit lower growth rates (Paasche 1971, Thompson et al. 1989, Turpin 1991, Clark and Flynn 2000).

### Dependence of NO_3_^−^ assimilation on carbon fixation

**Figure 7:**
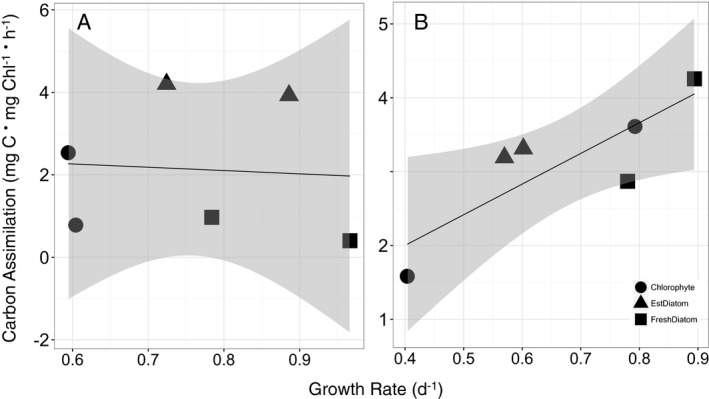
Carbon assimilation (mg C · mg Chl^−1^ · h^1^) as a function of cell‐specific growth rate (d^−1^) in six phytoplankton cultures growing on (A) NH_4_^+^ as the sole source of N for growth, or (B) NO_3_^−^ as the sole source of N for growth. Relationship between carbon assimilation and growth were estimated using regressions with slopes of 0.8 (NH_4_^+^, *r* ^2^ = 0.005, *P* = 0.89), and 4.2 (NO_3_^−^, *r* ^2^ = 0.702, *P* = 0.037). Grey shaded area denotes 95% confidence interval. Circles denote chlorophytes, triangles denote estuarine diatoms, and squares denote freshwater diatoms.

In contrast with growth rates, rates of mid‐day carbon assimilation were similar or greater when phytoplankton grew on NO_3_^−^ as a sole source of N compared with NH_4_^+^. In addition, relative differences in daytime carbon assimilation were correlated with relative differences in growth rates among species when they were grown on NO_3_^−^ as a sole source of N, but not with NH_4_^+^ (Fig. 7). One potential reason for these observations could be the tight regulation of NO_3_^−^ uptake by C‐fixation (Flores et al. 1983, 2005, Lara and Romero 1986, Turpin 1991). Because reduction in NO_3_^−^ to NH_4_^+^ is an energy intensive process, phytoplankton cells do not take up NO_3_^−^ in the absence of C‐fixation in order that cells lacking C skeletons for synthesis of amino acids will not carry out futile and costly NO_3_^−^ reduction (Turpin 1991, Flores et al. 2005, Mariscal et al. 2006, Sanz‐Luque et al. 2015). As a result, rates of NO_3_^−^ uptake and C‐fixation are tightly correlated, and occur during daytime when light is plentiful (Romero et al. 1985, Lara and Romero 1986). In contrast, more C may be fixed in darkness via phosphoenolpyruvate carboxylase in conjunction with anapleurotic C‐fixation by cells growing on NH_4_^+^ than by cells growing on NO_3_^−^ (Syrett 1956, Guy et al. 1989). This and other factors may contribute to a more moderate association of rates of NH_4_^+^ uptake and daytime C‐fixation (Lara and Romero 1986). In turn, this could explain the lack of correlation between growth rates and daytime C‐assimilation rates among different species when growing on NH_4_^+^, and overall faster growth rates of cells grown on NH_4_^+^, as they fix additional C at night‐time, compared with NO_3_^−^ grown cells. It is possible that the magnitude of these processes vary in a species‐specific manner, giving rise to the variability in growth rate differences with NH_4_^+^ and NO_3_^−^ observed in Table 3. Adding complexity to this picture is the fact that some species of phytoplankton are able to assimilate NO_3_^−^ at night time (i.e., Clark et al. 2002), thereby grow faster on NO_3_^−^, which may help explain the observation of slightly greater growth rates on NO_3_^−^ compared with NH_4_^+^ for *R. planktonicus* in the present experiments.

### Extrapolation of results from cultures grown on a single source of N to natural systems

While neither energetic considerations nor diel patterns in C and N assimilation may fully explain why most of the phytoplankton tested here exhibited faster rates of growth on NH_4_^+^ compared with NO_3_^−^, we question (i) how robust the patterns observed among species in these experiments are with variations in growth conditions (i.e., irradiance, temperature and N sufficiency) and (ii) how applicable the observed differences among species are to growth in natural systems where phytoplankton typically use more than one N source simultaneously. For example, N‐uptake measurements during monospecific blooms (>90% of community composition comprised of one species) demonstrate that phytoplankton take up two to three different forms of N at once (Maestrini et al. 1982, 1986, Berg et al. 1997, 2001, Kudela and Cochlan 2000, Collos et al. 2005). Moreover, culture studies investigating uptake of phytoplankton on a single source of N versus multiple sources demonstrate that total N‐uptake rate may be greater when multiple N sources are present at once compared with only one source (Lund 1987, Jauzein et al. 2008). This is consistent with our results with transient pulses of NO_3_^−^ or NH_4_^+^ in which C assimilation rates increased more when NH_4_^+^ was added (supplying cells with two different N sources) than when NO_3_^−^ was added (only one source of N present in culture) in NO_3_^−^grown cultures. This indicates that not only is total N‐uptake greater but C‐assimilation may also be greater when multiple sources of N are available. If that is the case, growth rates may also be higher in the presence of multiple N sources and the utility of measuring growth rates in phytoplankton grown on single sources of N to predict competition among species may be limited. For the future it would be interesting to compare growth rates on multiple versus single sources of N, and also to monitor the hierarchy of N‐uptake and depletion in the culture grown on multiple sources of N, to investigate differences among species that may be more applicable to natural conditions.

## Conclusions

Experiments with diatoms freshly isolated from the Sacramento River and Suisun Bay demonstrate that none are sensitive to NH_4_^+^ at concentrations up to 200 μmoles NH_4_^+^ · L^−1^, and some are not sensitive up to 1,000 μmoles NH_4_^+^ · L^−1^. Therefore, while manifestations of NH_4_^+^ toxicity are apparent in these data, onset of toxicity is unlikely to occur under typical environmental conditions, even when taking into consideration changes in pH and temperature. At environmentally relevant concentrations of N, we demonstrate that differences in growth rates calculated based on changes in cell abundance are detected in a number of species as a function of N source. Two diatom species and one chlorophyte grew significantly faster on NH_4_^+^ compared with NO_3_^−^, while a second chlorophyte grew significantly faster on NO_3_^−^ compared with NH_4_^+^. We show that in cases where growth rates are faster on NH_4_^+^ than they are on NO_3_^−^, the difference is not larger for chlorophytes compared with diatoms. This holds true for comparisons across a number of culture investigations suggesting that diatoms as a group will not be at a competitive disadvantage under natural conditions when NH_4_^+^ dominates the total N pool, and they will also not have a growth advantage when NO_3_^−^ is dominant, as long as N concentrations are sufficient. As demonstrated here, differences in growth rates among species, consistently higher in diatoms compared with the chlorophytes at 15°C–16°C, may play a greater role in determining competitive outcomes than variation in N source. These results have broad implications for evaluating phytoplankton community shifts in all estuarine systems where changes in N speciation are occurring, and particularly for high nutrient, low chlorophyll systems such as upper San Francisco Bay where resource managers are focusing on decreasing NH_4_^+^ concentrations specifically in an effort to boost growth of diatoms.

We sincerely thank Captains David Morgan and David Bell on the *R/V Questuary* and the other cruise participants for their support during the cruises in San Francisco Bay and the Sacramento River where we collected samples for phytoplankton isolations. We also thank three reviewers whose comments greatly improved this manuscript. This research was funded through the Interagency Ecological Program by the State and Federal Contractors Water Agency grant 13‐34 to GMB and the USDI Bureau of Reclamation award R14AP00053 to RMK. Further support was provided through the California Water Resources Control Board Award 22‐1509‐5082 to RMK, the Central Contra Costa Sanitary District award 42218 to GMB and 40969 to RMK, and the Sacramento Regional County Sanitation District award 90000094 to RMK.

## Supporting information


**Figure S1.** Map of San Francisco Bay, composed of four main subembayments: South Bay, Central Bay, San Pablo Bay, and Suisun Bay. The phytoplankton cultured for this study was isolated from Suisun Bay and the Sacramento River, a region denoted by the square.

**Figure S2.** Representative time course of changes in cell abundance (solid circle), Chl *a* (solid triangle), F_v_/F_m_ (solid square), NH_4_^+^ (open circle) during exponential growth in a culture (*Chlorella minutissima*) grown on low (20 μmoles NH_4_^+^ · L^−1^) and high (200 μmoles NH_4_^+^ · L^−1^) initial additions of NH_4_^+^. Initial and final cell abundances were 2.12 × 10^8^ ± 5.21 × 10^7^ cells · L^−1^ and 5.05 × 10^9^ ± 5.9 × 10^8^ cells · L^−1^, respectively. Initial and final Chl *a* concentration were 0.76 ± 0.7 μg · L^−1^ and 12.53 ± 5 μg · L^−1^, respectively. Increase in Chl *a* over course of the experiment was 16‐fold. Gray vertical line represents time point at which aliquots of the cultures were removed for determination of carbon fixation. Each data point represents the mean of three replicate cultures.

**Table S1.** Percent change in growth rates (relative to 20 μmoles NH_4_^+^ · L^−1^) with increasing concentrations of NH_4_^+^. Fifty percent decrease in the growth rates of *Asterionella ralfsii* and *Fragilaria capucina* was calculated to occur at NH_4_^+^ concentrations of 345 and ~762 μmoles · L^−1^, respectively.

**Table S2.** Regressions of growth rate (d^−1^) and Carbon assimilation (mg C · mg Chl^−1^ · h^−1^) as a function of medium N:P ratio (mol:mol) for each species.
