# Supplementary figures and images for: Variation in growth rate, carbon assimilation, and photosynthetic efficiency in response to nitrogen source and concentration in phytoplankton isolated from upper San Francisco Bay
Source: J Phycol. 2017 May 2;53(3):664–79. doi: 10.1111/jpy.12535 (PMC5518194; doi:10.1111/jpy.12535)

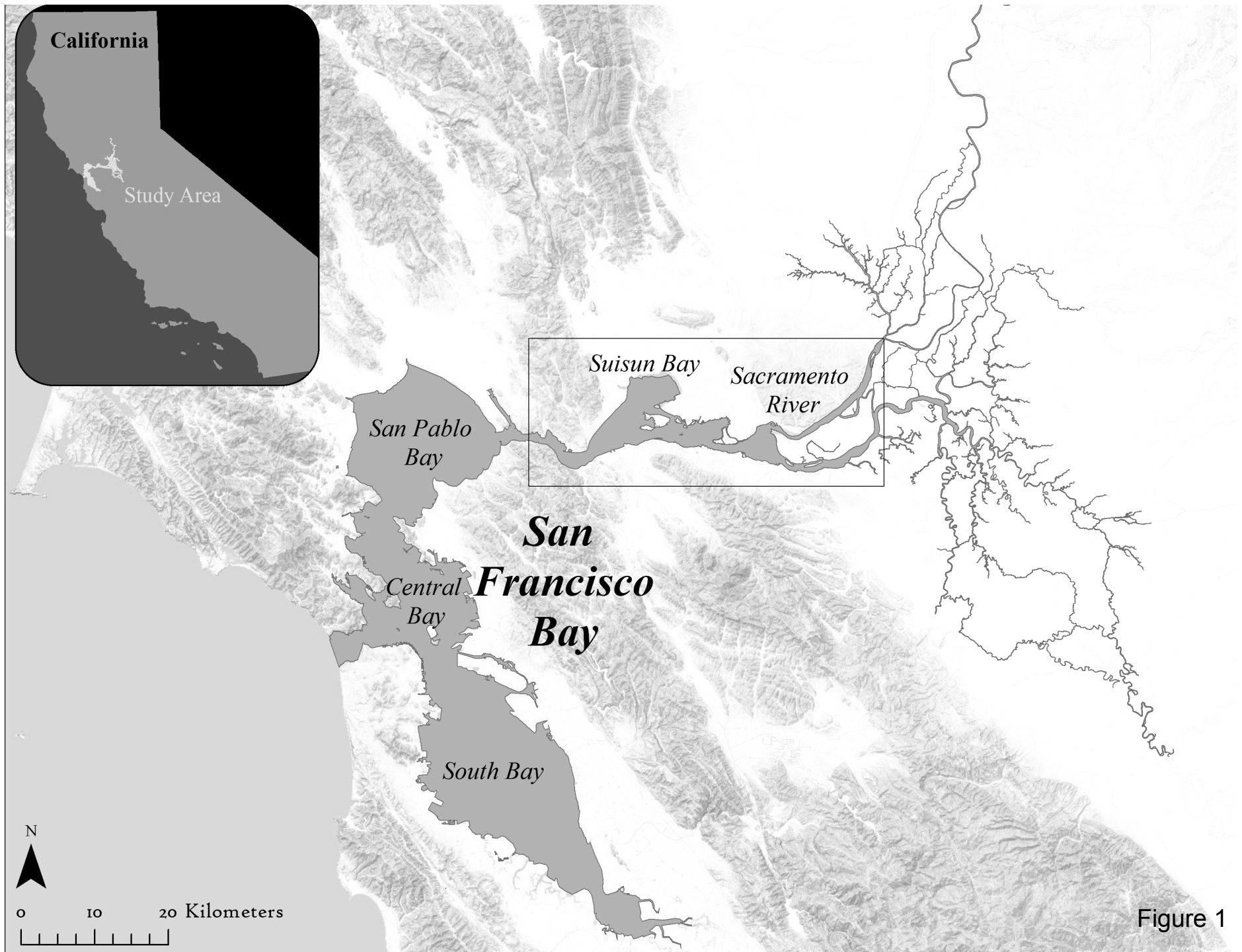

Supplement: Supplementary file 1 — Figure S1. Map of San Francisco Bay, composed of four main subembayments: South Bay, Central Bay, San Pablo Bay, and Suisun Bay. The phytoplankton cultured for this study was isolated from Suisun Bay and the Sacramento River, a region denoted by the square. [file JPY-53-664-s001.pdf]

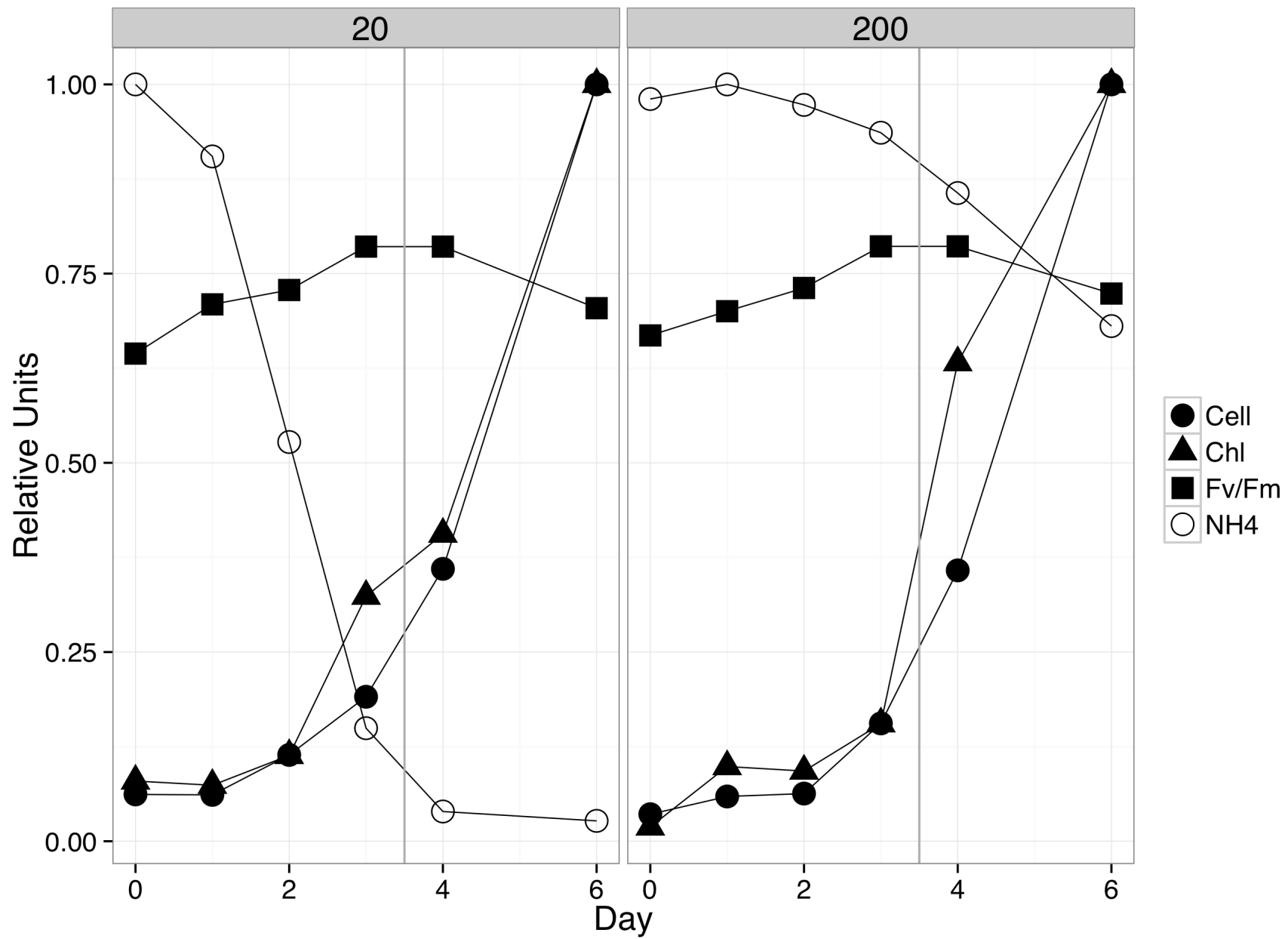

Supplement: Supplementary file 2 — Figure S2. Representative time course of changes in cell abundance (solid circle), Chl a (solid triangle), Fv/Fm (solid square), NH4 + (open circle) during exponential growth in a culture (Chlorella minutissima) grown on low (20 μmoles NH4 + · L−1) and high (200 μmoles NH4 + · L−1) initial additions of NH4 +. Initial and final cell abundances were 2.12 × 10^8^ ± 5.21 × 10^7^ cells · L−1 and 5.05 × 10^9^ ± 5.9 × 10^8^ cells · L−1, respectively. Initial and final Chl a concentration were 0.76 ± 0.7 μg · L−1 and 12.53 ± 5 μg · L−1, respectively. Increase in Chl a over course of the experiment was 16‐fold. Gray vertical line represents time point at which aliquots of the cultures were removed for determination of carbon fixation. Each data point represents the mean of three replicate cultures. [file JPY-53-664-s002.pdf]
