# Supplementary material for: Variation in growth rate, carbon assimilation, and photosynthetic efficiency in response to nitrogen source and concentration in phytoplankton isolated from upper San Francisco Bay
Source: J Phycol. 2017 May 2;53(3):664–79. doi: 10.1111/jpy.12535 (PMC5518194; doi:10.1111/jpy.12535)
Supplement: Supplementary file 3 — Table S1. Percent change in growth rates (relative to 20 μmoles NH4 + · L−1) with increasing concentrations of NH4 +. Fifty percent decrease in the growth rates of Asterionella ralfsii and Fragilaria capucina was calculated to occur at NH4 + concentrations of 345 and ~762 μmoles · L−1, respectively. [file JPY-53-664-s003.docx]

Table S1. Percent change in growth rates (relative to 20 µmoles NH_4_^+^ · L^-1^) with increasing concentrations of NH_4_^+^. Fifty percent decrease in the growth rates of *Asterionella ralfsii* and *Fragilaria capucina* was calculated to occur at NH_4_^+^ concentrations of 345 and ~762 µmoles · L^-1^, respectively.

| **NH_4_^+^ Concentration** | ***Chlorella*** | ***Entomoneis*** | ***Asterionella*** |
| --- | --- | --- | --- |
| 100 | 118.3 | 124.4 | 102.7 |
| 200 | 113.5 | 82.4 | 90.7 |
| 500 | 119.8 | 108.4 | 12.0 |
| 1000 | 120.7 | 101.4 | 0.0 |
| 3000 | 141.3 | nm | nm |
|  | ***Radiococcus*** | ***Thalassiosira*** | ***Fragilaria*** |
| 100 | 104.9 | 101.5 | 117.5 |
| 500 | 111.6 | 105.0 | 86.4 |
| 1000 | 146.0 | 113.3 | 20.7 |
| 3000 | 116.2 | 65.2 | 27.6 |

nm=not measured
