# Supplementary material for: Variation in growth rate, carbon assimilation, and photosynthetic efficiency in response to nitrogen source and concentration in phytoplankton isolated from upper San Francisco Bay
Source: J Phycol. 2017 May 2;53(3):664–79. doi: 10.1111/jpy.12535 (PMC5518194; doi:10.1111/jpy.12535)
Supplement: Supplementary file 4 — Table S2. Regressions of growth rate (d−1) and Carbon assimilation (mg C · mg Chl−1 · h−1) as a function of medium N:P ratio (mol:mol) for each species. [file JPY-53-664-s004.docx]

Table S2. Regressions of growth rate (d^-1^) and Carbon assimilation (mg C · mg Chl^-1^ · h^-1^) as a function of medium N:P ratio (mol:mol) for each species.

| **Species** | **Regression Param** | **Growth vs N:P** | **C assim vs N:P** |
| --- | --- | --- | --- |
| ***Chlorella*** | Slope | 0.001 | -0.038 |
|  | r^2^ | 0.15 | 0.09 |
|  | p | 0.02 | 0.08 |
|  | F Statistic | 6.0 | 3.3 |
|  | Df | 34 | 34 |
| ***Radiococcus*** | Slope | -0.000 | -0.015 |
|  | r^2^ | 0.00 | 0.03 |
|  | p | 0.88 | 0.35 |
|  | F Statistic | 0.0 | 0.9 |
|  | Df | 28 | 28 |
| ***Entomoneis*** | Slope | 0.001 | 0.120 |
|  | r^2^ | 0.00 | 0.11 |
|  | p | 0.76 | 0.07 |
|  | F Statistic | 0.1 | 3.4 |
|  | Df | 28 | 28 |
| ***Thalassiosira*** | Slope | -0.001 | 0.040 |
|  | r^2^ | 0.03 | 0.14 |
|  | p | 0.33 | 0.06 |
|  | F Statistic | 1.0 | 3.8 |
|  | Df | 28 | 28 |
| ***Asterionella*** | Slope | 0.033 | -0.11 |
|  | r^2^ | 0.16 | 0.01 |
|  | p | 0.09 | 0.70 |
|  | F Statistic | 3.2 | 0.1 |
|  | Df | 16 | 16 |
| ***Fragilaria*** | Slope | -0.007 | -0.13 |
|  | r^2^ | 0.05 | 0.02 |
|  | p | 0.38 | 0.61 |
|  | F Statistic | 0.8 | 0.3 |
|  | Df | 16 | 16 |
